# Restoring dignity through joint care - A decade of project Gift of Disability Alleviation (GODA) in Pakistan

**DOI:** 10.12669/pjms.42.(ICON26).15692

**Published:** 2026-04

**Authors:** Muhammad Amin Chinoy, Umar Burney, Mansoor Ali Khan, Saima Ali

**Affiliations:** 1 Muhammad Amin Chinoy, Indus Hospital and Health Network, Korangi Crossing, Karachi, Pakistan; 2 Umar Burney, MD. President and Founder, Project GODA. Indus Hospital and Health Network, Korangi Crossing, Karachi, Pakistan; 3 Mansoor Ali Khan, Section Head of Orthopaedics, Aga Khan University Hospital, Karachi, Pakistan; 4 Saima Ali, Head of Emergency Medicine, Indus Hospital and Health Network, Korangi Crossing, Karachi, Pakistan

Joint diseases are a leading cause of disability worldwide with an estimated 528 million people globally living with musculoskeletal disorders, including osteoarthritis (OA), rheumatoid arthritis (RA) and related conditions.^1^ South Asia bears a disproportionate burden^2^ with Pakistan showing an increase in the prevalence of OA from 2.85 million cases in 1990 to 8.5 million cases in 2021, reflecting an ageing population and unmet needs in preventive care.^3^ Over the same period, RA-related disability-adjusted life years (DALYs) nearly doubled.^4^ Road traffic accidents continue to cause long-term disability, especially among the economically disadvantaged for whom access to specialized care like joint replacement surgery is limited or prohibitively expensive.^5^

In the face of this challenge, Project GODA (Gift of Disability Alleviation), an initiative of the Indus Hospital and Health Network (IHHN), has emerged as a beacon of hope over the past decade. Beginning as a small pilot collaborative mission between healthcare professionals from the United States and Pakistan, Project GODA has grown steadily and is now in its 8^th^ year of providing life-changing, free of cost surgeries to patients suffering from advanced joint diseases and injuries.^6^ In total, the volunteer team of orthopedic surgeons, nurses, anesthetists, physical therapists and volunteers from major implant manufacturers; hand-in-hand with their counterparts at IHHN, have performed more than 700 surgeries till date. The scope of the annual surgical camps has expanded significantly from 120 procedures done in 2023 to 300 patients in 2025 where the teams performed 243 joint replacements, 25 spinal surgeries and 31 anterior cruciate ligament (ACL) reconstructions, with zero cost to the patients.^7^

This project fosters an environment of mutual learning and trust where local surgical teams and patients gain exposure to advanced techniques and the visiting teams benefit from local knowledge and support. The project also ensures continuity of care where patients transition seamlessly into IHHN’s regular outpatient system for rehabilitation and ongoing evaluation. The project’s multi-city approach, conducting camps at Karachi, Lahore and Muzaffargarh, among others, has helped in bringing tertiary care to underserved regions, aligning with broader public health goals of reducing regional disparities in healthcare access. The ripple effects are considerable as a successful joint surgery can enable a patient to return to work, reduce household poverty and relieve the caregiving burden. In a country where musculoskeletal disorders contribute significantly to years lived with disability, Project GODA serves as a high-impact, small-scale intervention that transforms disability into renewed productivity.

Project GODA’s decade of accomplishments highlights what is possible when compassion and cross-border collaboration converge on a common cause. It exemplifies how local leadership with their partners can bolster a developing country’s healthcare system by addressing gaps in care. We call on healthcare leaders, policy-makers and philanthropists to recognize and support initiatives like Project GODA. This support can take many forms; formalizing exchange programs for surgical teams, encouraging corporate social responsibility contributions from medical device companies, and allocating public or donor funding to scale up free-of-cost surgical camps. We also urge local medical institutions to partner in knowledge transfer so that more Pakistani surgeons are trained in advanced joint repair techniques, building local capacity for the long term. By investing in such collaborative efforts, Pakistan’s health sector can make tangible strides toward alleviating disability and ensuring that the ability to walk, work, and live free of pain is not a luxury but a right accessible to all.
